# Reasons and Outcomes of Pharmacy-Initiated Communication with Medical Practices—A Flashmob Study in Germany

**DOI:** 10.3390/healthcare14121602

**Published:** 2026-06-06

**Authors:** Paul Boack, Annett Braesigk, Franka Uhlendorff, Sophie Karl, Markus Bleckwenn, Anne Schrimpf

**Affiliations:** Institute for General Practice, Faculty of Medicine, Leipzig University, 04109 Leipzig, Germany; annett.braesigk@medizin.uni-leipzig.de (A.B.); markus.bleckwenn@medizin.uni-leipzig.de (M.B.); anne.schrimpf@medizin.uni-leipzig.de (A.S.)

**Keywords:** interprofessional relations, community pharmacy services, physician-pharmacist communication, prescription drugs, drug shortages, primary health care, health services research

## Abstract

**Highlights:**

**What are the main findings?**

- Prescription-related contacts are mainly driven by drug shortages and formal errors.
- Pharmacists’ clinical expertise is rarely the primary reason for physician contact.

**What are the implications of the main findings?**

- Fewer contact attempts and accepted recommendations increase satisfaction.
- Case resolution is faster in communication with general practices.

**Abstract:**

**Background/Objectives**: Effective communication between medical practices and community pharmacists is essential for safe and efficient outpatient care, yet quantitative evidence from the perspective of community pharmacies regarding the frequency, content, and efficiency of routine pharmacist–physician communication in Germany is limited. This study aimed to investigate, from the perspective of community pharmacies, the reasons for and outcomes of initiating contact with medical practices following the submission of patient prescriptions in routine outpatient care. **Methods**: In this observational study, 45 community pharmacies in Leipzig and the surrounding region (Germany) documented simultaneously all contacts with medical practices related to prescriptions over a 14-day period (November 2023) using a standardized documentation form. Data included reasons for contact, communication channels, number of contact attempts, outcomes, satisfaction ratings, and case duration. Multivariable linear regression was used to identify factors associated with satisfaction with contact outcomes, and ordinal regression to examine determinants of case duration. **Results**: In total, 498 documented contacts were analyzed. The most frequent reasons for contact were drug availability issues and formal or content-related prescription errors. Consultations related to drug interactions or contraindications were rare. Overall satisfaction with contact outcomes was high, but lower satisfaction was associated with repeated contact attempts, non-acceptance of pharmacists’ recommendations, and contacts related to formal prescription errors. Case resolution was faster when fewer contact attempts were required and when communicating with general practices rather than specialist practices. **Conclusions**: Pharmacy-initiated communication with medical practices in outpatient care is largely driven by administrative and logistical issues rather than clinical consultations. Improving prescription quality, enhancing transparency of drug availability, and strengthening efficient communication pathways may reduce workload and increase satisfaction.

## 1. Introduction

Collaboration and effective communication between physicians and pharmacists are essential to delivering high-quality patient care. In primary care in particular, pharmacists’ expertise remains underutilized and often undervalued [1,2]. Evidence from intervention studies demonstrated that stronger physician–pharmacist collaboration can improve both clinical and system-level outcomes [3,4]. Reported benefits include improved access to care in underserved regions, enhanced disease management, reductions in medication-related hospitalizations and mortality, decreased polypharmacy, and lower medication costs [3,4,5,6,7,8,9,10]. While clinical pharmacy is well established within multidisciplinary teams in hospitals [11,12], comparable collaborative structures between physicians and community pharmacists in outpatient care remain limited and continue to require systematic development [1,2,13].

Regardless of how extensively pharmaceutical expertise is integrated into patient care, regular communication between medical practices and community pharmacies is indispensable in outpatient settings, for example to address supply shortages, potential drug interactions, or prescription inconsistencies [14]. Effective interprofessional communication and cooperation are therefore essential to maintaining continuity and quality of care [1,15]. International research has identified several factors that facilitate successful collaboration, including physical proximity between professionals [1,16,17], appreciative and trusting communication, and a shared understanding of roles [15,18,19]. Conversely, a lack of time and staff, unclear delineation of responsibilities and roles, uncertainty regarding financial compensation, poor accessibility of medical practices, and the lack of standardized communication structures have been shown to hinder effective physician-pharmacist interaction [2,9,14,16,20,21,22].

At the same time, community pharmacies in Germany are expanding their public-health roles beyond traditional dispensing and compounding services [6,23,24], in part to reinforce their position within local healthcare systems in the face of increasing online competition. In routine practice, however, physicians’ acceptance of these services has remained inconsistent [25]. Furthermore, under the legal and regulatory frameworks in force in Germany, the exclusive authority of physicians to make prescribing decisions (“physician’s prerogative”) substantially limits the ability of community pharmacies to independently modify medication regimens, for example in response to drug shortages or other drug-related problems [26,27]. Despite ongoing discussions regarding a potential expansion of pharmacists’ responsibilities under the proposed Pharmacy Supply Development Act [28], it remains unclear whether the full professional competencies of community pharmacies are being adequately utilized within outpatient healthcare in Germany.

Against this background, communication between community pharmacies and medical practices is essential in outpatient care, particularly when pharmacies identify prescription-related problems requiring clarification. The aim of this study was therefore to investigate, from the perspective of community pharmacies, the reasons for initiating contact with medical practices following the submission of patient prescriptions in routine outpatient care, and to characterize the modalities and outcomes of these interactions. For this purpose, pharmacies in a major German city and its surrounding area were asked to document each communication event with medical practices using a standardized paper-based documentation form. Specifically, pharmacies were asked to assess the frequency of contact attempts, the communication channels used, the actors involved in each interaction, and the reasons for initiating contact. In addition, they were asked to evaluate the perceived quality, efficiency, and outcomes of these exchanges. Furthermore, the study sought to examine potential factors influencing satisfaction with the outcome of each contact, including the medical specialty involved, the underlying reason for the interaction, or the extent to which the pharmacy’s recommendation was accepted. Lastly, as the duration of each contact directly affects workflow efficiency, resource allocation, and the timeliness of patient care, this study also examined which factors most strongly influenced the length of these interactions.

## 2. Materials and Methods

### 2.1. Sampling and Research Design

The survey was conducted in the city of Leipzig and the surrounding area in Saxony, Germany. The study was designed as a “flashmob study”, a research approach characterized by the rapid and simultaneous collection of real-world data by a large number of participants within a short predefined time period. This methodology enables the capture of routine practice patterns under everyday conditions while minimizing long-term changes in behavior or external influences during the study period [29]. Data were collected within 14 days from 6 November 2023 to 19 November 2023. Pharmacies were invited to participate in this study on a voluntary basis and to complete the standardized documentation form each time they had contact with a medical practice regarding a prescription.

Pharmacies were initially invited to participate by post. The email and postal addresses of the invited pharmacies were obtained from the publicly accessible pharmacy finder on the website aponet.de, which is also recommended by the German Association of Pharmacists (ABDA) as a resource for locating pharmacy contact information. An invitation was sent to all pharmacies in the Leipzig area (including the city and its surrounding rural region) that were listed in the database. Non-responders received an email reminder after three weeks, followed by a final reminder via telephone. In total, 192 pharmacies were contacted, of which 45 agreed to participate, yielding a response rate of 23.4%. No pre-defined inclusion or exclusion criteria were applied.

General and demographic characteristics of the participating pharmacies were collected as part of a short preliminary survey before the study documents were distributed. This included the postal code, a self-assessment of the respective catchment area, the average number of customers per day, and the structure of the workforce. Sample characteristics of participating pharmacies can be found in Table 1.

**Figure 1:**
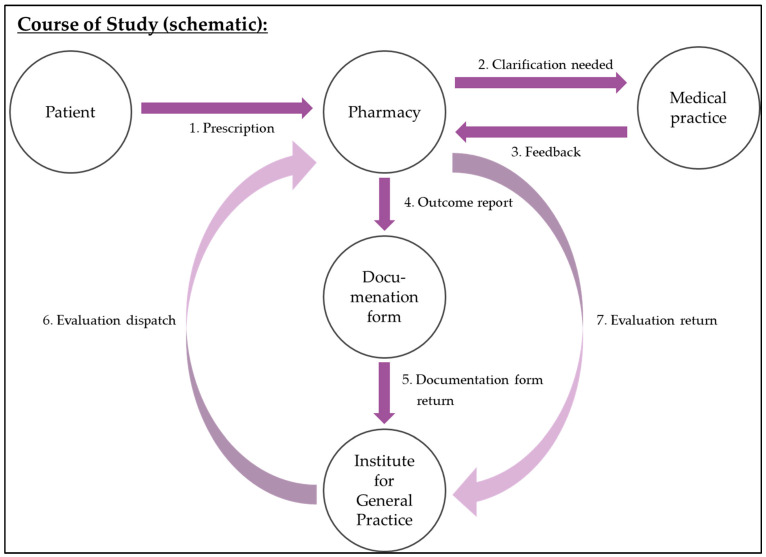
Flowchart of the study procedure.

Each participating pharmacy received 100 documentation forms to document contacts with medical practices, along with a link to a training video demonstrating how to complete the documentation form. Pharmacies were pseudonymized using unique IDs, and all case documentation was completed anonymously. After a 14-day documentation period, pharmacies were asked to return the completed forms. Subsequently, they were invited to complete an evaluation of the study procedure. The flowchart of the study procedure can be found in Figure 1.

### 2.2. Standardized Documentation Form

The documentation form was developed by an interdisciplinary research team (medical and social scientists, pharmacists) at the Institute for General Practice at Leipzig University in a multi-stage revision process. The content of the documentation form was based on preliminary interviews with three pharmacists and two general practitioners (GPs) and an extensive literature search. Each version of the documentation form was continuously discussed with professional representatives. The final version was subjected to a think-aloud pre-test [30] with three pharmacists to identify problems or misunderstandings related to each item. After pre-testing, the documentation form was adapted and further developed. The final version of the documentation form can be found in Supplementary Material S1.

The documentation form covered the following topics: (1) contact details (e.g., date and time of contact, specialization of practice, postcode of practice, means of contact, number of contact attempts), (2) reasons for contact (e.g., interactions, contraindications, dosage/application, plausibility, availability, names of medicinal products), (3) contact outcomes (e.g., accessibility of practice, recommendation accepted, perceived satisfaction with contact, processing time of the request), (4) free text field for additional comments on the contact and its outcome.

### 2.3. Study Evaluation

Participating pharmacies were asked to complete an evaluation of the study procedure after the 14-day study period. Non-responding pharmacies were followed up by telephone to ensure the return of all evaluation forms. This procedure resulted in a 100% response rate.

The evaluation covered the following topics: (1) assessment of integration into daily pharmacy practice, (2) evaluation of the documentation form (e.g., relevance of content, time and personnel requirements), and (3) occurring problems during the study period. The final version of the evaluation is provided in Supplementary Material S2.

### 2.4. Coding of Free Text Entries

Participating pharmacies were asked to indicate in a free-text field, if needed, additional comments on the contact and its outcome. The statements were originally collected in German. Example quotes were translated into English and slightly edited for readability. For analysis, free text entries were independently coded by two authors of this study. Categories were derived inductively during the coding, either indicating a major category or a subcategory. The assignments were compared and differences in coding were discussed until inter-coder agreement was reached for each discrepancy.

### 2.5. Statistical Analysis

All statistical analyses were carried out using IBM SPSS Statistics 29 (Armonk, NY, USA) with a two-sided α-level of 0.05. For descriptive statistics, missing values in single variables were considered by presenting frequencies as % (n/nvalid). Continuous variables were presented as mean (M) ± standard deviation (SD).

A multiple linear regression model using Entry method was conducted to calculate potential associations between the satisfaction with the outcome of the contact (rating scale from 1 to 10) and independent variables such as specialization of the practice (GP vs. specialist), number of contact attempts, duration of the conversation, acceptance of the pharmacy’s recommendation, and reasons for establishing contact. The assumptions of linearity, residual normal distribution, residual variance homogeneity, residual independency, and no multicollinearity were tested by scatter and p-p plots, Durbin–Watson statistics, and variance inflation factor (VIF). Cook’s distance was used to detect outliers.

Lastly, a multivariate ordinal regression model with logit link function was used to examine the association between duration of the entire case (1 < 60 min; 2 = 1–12 h; 3 = 13–24 h; 4 > 24 h) and specialization of the practice (GP vs. specialist), accessibility (contact made & reply received; no one could be reached), and reasons for establishing contact. The strength of association was reported as 95% confidence intervals (CI).

## 3. Results

### 3.1. Sample Characteristics of Contacted Practices and Means of Contact

In total, 498 documentation forms were filled out and were eligible for analysis. Percentages, means, and standard deviations for sample characteristics—details of the contacted practices and means of contact—can be found in Table 2.

### 3.2. Reasons for Establishing Contact

Main reasons for establishing contact with medical practices were drug availability issues (52.2%) and issues coded as “others” (35.9%), which mainly related to formal or content errors on the prescriptions as well as a need for additional information. Percentages for all reasons can be found in Table 3. A complete overview of the prescribed drugs and medical products can be found in Supplementary Material S3.

### 3.3. Outcome of and Satisfaction with Contact

Most pharmacies reported that their recommendations for addressing the problem were accepted by the practices (86%). Overall, satisfaction with the outcome of the contact (M = 8.3) as well as the perceived pleasantness of the interaction (M = 8.7) were both rated very highly on a 1–10 numerical rating scale. Further details on contact outcomes are provided in Table 4.

A multiple linear regression was calculated to assess relationships between satisfaction with the outcome of the contact and independent variables such as specialization of the practice (GP vs. specialist), number of contact attempts, duration of the conversation, acceptance of the pharmacy’s recommendation, and reasons for establishing contact. The model explained 39% of the variation in satisfaction with the outcome of the contact (F(10, 261) = 17.655, *p* < 0.001; Table 5). In this model, satisfaction was highest when fewer contact attempts were required, when the pharmacy’s recommendation was accepted, and when the reason for establishing contact did not fall under the “other” category, which primarily consisted of formal or content errors in the prescriptions.

An ordinal regression was calculated to assess relationships between the duration of the entire case and independent variables such as specialization of the practice (GP vs. specialist), number of contact attempts, accessibility, and reasons for establishing contact (Table 6). The assumption of proportional odds was confirmed (χ^2^(18) = 11.656, *p* = 0.864). The model accounted for a significant proportion of variance (Nagelkerke R^2^ = 0.199). The model indicated that the duration was shorter for contacts with GP practices and for fewer contact attempts. Reasons for establishing contact were not related to duration.

### 3.4. Free Text Comments

Participating pharmacies could use a free text field to provide additional details about the cases, which was used in n = 292 cases. Further details of the free text analysis are presented in Table 7. The statements indicated that issues with the prescriptions resulted in additional effort for all parties involved.

*“Discontinued after 24 h without a solution. The patient should address the matter in person at the practice.”*

*“Positive in-person interaction with the physician; the previous product was prescribed. For future prescriptions, the correct successor preparation should be issued. However, this did not work, as the prescription was again incorrect, requiring renewed contact.”*

*“The patient needs to begin therapy urgently. Although a nurse works in the practice, no one responds to contact attempts, as patients are seen on Fridays by appointment only. However, the prescription would need to be amended for the alternative treatment options. The patient was not willing to return to the practice again and therefore could not be provided with care.”*

In most cases, patient care was maintained with prescriptions either issued as originally intended or replaced with an appropriate alternative.

*“The patient had already visited several pharmacies regarding availability. We identified an alternative and requested a prescription.”*

*“The physician prescribed a dosage that was too low (150 µg instead of 175 µg). The patient always receives 175 µg; a new prescription was obtained from the practice.”*

*“Friendly telephone conversation; after explanation, the issue was understood and the proposed solution was gratefully accepted, allowing a rapid outcome and satisfaction for all parties (physician–pharmacy–patient).”*

The poor availability of the practices was a recurrent theme in the statements.

*“Multiple telephone calls required; attempts over three days were necessary before achieving success.”*

*“Call was answered only on the fourth attempt; the physician fundamentally rejects the pharmacy’s concerns and shows no willingness to compromise.”*

*“There were multiple unsuccessful telephone calls despite designated phone consultation hours. Contact was then made with the nurse, who promised a return call within the next hour. The return call did occur, but not at 09:00 as indicated; it was made only at 16:30, which was far too late.”*

*“Only voicemail was available; the pharmacy was unable to make contact. The patient had to visit the practice in person to obtain a new prescription.”*

### 3.5. Study Evaluation

After study completion, participating pharmacies were asked to evaluate the study and provided materials. Staff shortages and large numbers of customers were rated as the main barrier to documenting the cases. Further details are provided in Supplementary Material S4.

## 4. Discussion

By using a standardized documentation form, pharmacies documented each communication event with medical practices. The study showed that contact was most frequently initiated due to drug availability issues and various formal or content-related prescription errors. Although interactions were generally rated as pleasant and satisfactory, factors such as repeated contact attempts, non-acceptance of the pharmacy’s recommendation, and prescription errors were associated with lower satisfaction. Moreover, the total duration of a case was shorter when the contacted practice was a general practice and when fewer contact attempts were required.

**Reasons for establishing contact.** The main finding of our study was that contact was predominantly initiated due to drug availability and formal prescription errors, whereas consultations concerning drug interactions or contraindications, and thus core aspects of medication safety, played only a minor role. Although the literature consistently emphasized pharmacists’ core competencies as medication experts and their potential contributions within multidisciplinary healthcare teams [7,8,9,13], our findings indicate that such expertise-driven inquiries after prescription submission are rarely the primary reason for contact, suggesting that pharmacists’ professional knowledge might be underutilized. As the legislation in force in Germany at the time of the study substantially limited the ability of community pharmacies to independently modify medication regimens, any adjustment to a prescription required authorization by the prescribing physician [26,27]. Our results are in line with a recent study, which reports that physician-pharmacist contact most commonly occurs in the correction of medical prescription errors, while consultations regarding drug selection, side effects, drug interactions, or medical devices are reported only rarely [2].

The predominance of drug availability issues as the primary driver for pharmacists contacting physicians highlights a significant structural inefficiency within the healthcare system. As drug shortages are expected to become more frequent due to manufacturing bottlenecks, economic pressures, and a lack of diversified production sites [31], pharmacists might be compelled to assume the role of supply chain managers rather than clinical consultants [32]. To mitigate the burden associated with drug availability for both pharmacists and physicians, GPs in England have proposed the implementation of technical solutions that allow prescribers to identify medication availability prior to issuing a prescription [17].

Likewise, the high frequency of contacts related to formal prescription errors, such as missing signatures or incomplete information, results in a communication pattern characterized by high volume but limited clinical value. As other studies reported similar prescription issues [2,33], the widespread occurrence of these errors suggests a need for interventions. A study conducted in Germany highlights the importance of structured communication and the harmonization of key procedural processes, including targeted training initiatives led by pharmacists to improve prescription accuracy and reduce the recurrence of formal errors [16].

**Outcome of and satisfaction with the contact.** Our results indicate that pharmacist-medical practice contact was perceived as predominantly positive with respect to both outcomes and interpersonal experience. However, the literature presents inconsistent assessments of interactions between pharmacies and medical practices: while some studies highlight largely positive interprofessional collaboration [21], others report more negative experiences [2], suggesting that additional contextual or structural factors may influence these perceptions.

We further found that satisfaction with the outcome was particularly high when pharmacist recommendations were accepted, fewer contact attempts were required, and a smaller proportion of contacts fell into the “Other” category, primarily reflecting formal or content-related prescription errors as reasons for initiating contact. These results highlight the importance of logistical efficiency. Repeated attempts to establish contact are frequently cited as a source of frustration and unnecessary time expenditure for pharmacy staff [13,16,17,21]. Our findings also suggest that a lower proportion of formal or content-related reasons for initiating contact was associated with better ratings. Such errors are often perceived as avoidable and unreflective of professional diligence, requiring reactive problem-solving rather than proactive, clinically driven collaboration [20]. Consequently, while clinical consultations allow for an exchange on equal professional footing, administrative corrections are often viewed as time-consuming burdens [16,21]. Further, the positive association between outcome satisfaction and acceptance of pharmacist recommendations is likely rooted in the psychological validation of professional expertise. When a medical practice accepts a pharmacist’s suggestion, it fosters a sense of mutual respect and effective cooperation, whereas rejection can lead to feelings of futility [1,16,21]. This finding aligns with broader literature identifying trust and mutual respect as fundamental pillars of successful healthcare collaborations [19,20,34].

**Duration of contact and effort.** Our findings indicate that the majority of pharmacy-initiated communication occurs with general practices (55.1%). Consistent with previous observations [16], the most favorable time to reach medical practices was during the late morning hours, shortly before the customary lunch break (mean time: 11:27 ± 2:31 a.m.). Telephone calls were the preferred mode of communication in our study. Previous research has suggested that telephone-based communication may be associated with more successful and timely resolution of prescription-related issues [35], which may partly explain its predominant use. The documented cases in our study required on average two contact attempts. Consistently, pharmacies reported in optional free-text responses that additional contacts during the prescription process, particularly those necessitated by formal errors, substantially increased the workload for all parties involved, including patients, pharmacies, and medical practices. This observation highlights a structural challenge, as previous research has repeatedly demonstrated that both medical practices and pharmacies operate under considerable time constraints [8,9,14,17,21].

We further examined factors influencing the overall duration of case resolution. We found that a shorter processing time was primarily associated with a lower number of contact attempts. Difficulty in reaching physicians has been described previously as a major source of frustration in pharmacist-physician communication, leading to delays in patient care [16,17] and high workload for pharmacies [35]. We further found that the duration of the case was shorter when pharmacies communicated with general practices compared with other medical specialists, whereas the specific reason for initiating contact did not significantly affect case duration. One possible explanation is that collaboration with general practices benefits from well-established professional relationships, which are fostered by more frequent interactions and a generally higher level of routine exchange [1]. The absence of an association between the reason for contact and processing time may indicate that pharmacies manage most issues efficiently within their professional scope. This aligns with previous findings showing that physicians value concise, solution-oriented communication, particularly when pharmacies propose clear and actionable recommendations [21,22].

**Limitations.** Several limitations should be considered when interpreting the results of this study. First, the geographic scope was limited to the city of Leipzig and its surrounding areas in Saxony, Germany. Although the sample included both urban and rural pharmacies, the results may not be fully representative of all community pharmacies in Germany or generalizable to other regions. Differences in regional healthcare structures, prescribing patterns, availability of medical services, implementation of digital prescribing systems, and interprofessional communication practices may influence the frequency and nature of pharmacist-physician interactions. For instance, the prior implementation of regional pilot projects such as ARMIN (“Arzneimittelinitiative Sachsen-Thüringen”), may have resulted in pharmacies in Leipzig being particularly sensitized to issues of interprofessional communication and potentially more proactive in this regard than pharmacies in other regions [24].

In line, the participation rate of 23.4% (45 out of 192 pharmacies) might have introduced a selection bias. Pharmacies with a particular interest in interprofessional collaboration or communication with medical practices may have been more likely to participate, potentially limiting the representativeness of the sample. Consequently, the findings may not fully reflect the experiences and communication practices of all community pharmacies in Germany.

Further, in line with our research question, the study design was unidirectional, capturing only contacts initiated by pharmacies toward medical practices. Therefore, communication events initiated by medical practices towards pharmacies were not recorded and could not be included in the analysis. Future studies should also consider the perspectives of medical practices.

Although pharmacies were instructed to document every contact with a medical practice during the 14-day period, it is likely that not all contacts that occurred during the study period were documented. Possible reasons include staff shortages and the particularly high workload during the study period in November, which pharmacies identified as the main barriers to participation in the study, as indicated by the study evaluation. This suggests a potential under-reporting bias, where more complex or time-consuming cases might have been omitted during peak hours, potentially skewing the results toward less intensive interactions.

In addition, the documentation form is not a valid scale as we did not develop and assess several items measuring a construct related to the modalities, efficiency, and quality of interaction between pharmacies and medical practices, but rather investigated single-item responses. Single-item responses were chosen over scales to reduce the length of the documentation form and, hence, increase willingness to participate in a population with time constraints. However, studies showed that single-item responses might be as reliable as multiple-item scales, especially for less complex constructs, for example [36,37].

Finally, the study period in November 2023 coincided with the transition phase toward mandatory use of electronic prescriptions (e-prescriptions) in Germany. At that time, paper-based prescriptions were still widely used and therefore predominated in the present study, whereas e-prescriptions were still in a testing phase. Consequently, the results may no longer fully reflect the current situation, in which some barriers associated with paper-based processes may have been reduced, while new challenges related to digitization may have emerged. Against this background, repeating the study at a later point in time would be valuable to assess changes in interprofessional communication under fully implemented digital prescribing conditions.

**Implications.** The findings of this study have several implications. First, the predominance of pharmacy-initiated contacts related to drug availability and formal prescription errors indicates a misalignment between pharmacists’ clinical competencies and their role in interprofessional communication. When interactions are largely administrative, pharmacists risk being perceived as intermediaries rather than clinical partners [25]. This underscores the need for mutual understanding of professional roles, which could be fostered through joint training and continuing education [20,24]. Further, the high volume of contacts related to drug shortages highlights the urgency of system-level solutions to address supply chain transparency. Providing prescribers with real-time information on medication availability at the point of prescribing could substantially reduce avoidable contacts, decrease delays in patient care, and alleviate workload pressures for both pharmacies and medical practices. In line, the frequent occurrence of formal prescription errors points to persistent deficiencies in prescribing processes. Standardized prescription formats, clearer procedural guidelines, and targeted interprofessional training, potentially led by pharmacists, could reduce avoidable errors and the resulting communication burden [38,39]. Addressing these frequent reasons for contact may enhance overall satisfaction with interprofessional collaboration and free time resources for all healthcare providers.

Second, the associations between satisfaction, acceptance of pharmacist recommendations, and fewer contact attempts emphasize the importance of mutual trust, respect, and efficient communication pathways. Structured communication strategies and defined points of contact within medical practices may help minimize repeated attempts and foster more effective collaboration. Third, the shorter case resolution times observed when communicating with general practices suggest that continuity of collaboration and established professional relationships facilitate efficiency. This finding implies that fostering regular, routine exchange between pharmacies and medical practices may also improve communication efficiency with other medical specialties.

## 5. Conclusions

This study provides detailed insight into the nature, drivers, and perceived quality of pharmacy-initiated communication with medical practices in routine outpatient care. Although interprofessional interactions were generally experienced as pleasant, they were predominantly triggered by drug availability problems and formal or content-related prescription errors rather than by clinically driven consultations. This pattern points to a structural imbalance in which pharmacists’ core competencies as medication experts might be underutilized. Satisfaction with communication was closely linked to logistical efficiency, particularly the number of contact attempts and the acceptance of pharmacist recommendations, emphasizing the importance of mutual trust, professional recognition, and well-functioning communication pathways. Shorter case resolution times in interactions with general practices further suggest that established professional relationships and routine exchange facilitate more efficient collaboration. Addressing these structural challenges could shift pharmacist–physician interactions toward more clinically meaningful collaboration, reduce workload for all parties involved, and ultimately support safer and more efficient patient care.

## Figures and Tables

**Table 1:** Sample characteristics of participating pharmacies.

n	45
Postal code of pharmacies	
City of Leipzig	32 (71.1%)
Surrounding suburban/rural area	13 (28.9%)
Catchment area *	
City	32 (71.1%)
Small town	15 (33.3%)
Rural	4 (8.9%)
Number of customers per day	
≤150	15 (33.3%)
151–250	14 (31.1%)
251–350	6 (13.3%)
≥351	1 (2.2%)
No answer	9 (20.0%)
Employees	
Pharmacists	2.9 ± 1.3 (range 1–6)
Pharmacy engineers	1.5 ± 0.5 (range 1–2)
Pharmaceutical–technical assistants	3.6 ± 2.6 (range 1–15)
Pharmaceutical–commercial assistants	1.1 ± 0.3 (range 1–6)
Others	1.8 ± 1.3 (range 1–6)

Note. Values represent n, percentage of valid cases (%), mean, standard deviation, and range. * = multiple responses possible. Pharmacy engineer = Apprenticeship program in the former German Democratic Republic (GDR), involving responsibilities falling between those of a pharmacy technician and a pharmacist; profession is being phased out.

**Table 2:** Sample characteristics of the cases.

n	498
**Details of contacted practices**	
Postal code of practices	
City of Leipzig	335 (67.7%)
Surrounding suburban/rural area	160 (32.3%)
No answer	3
Specialization of practice	
General practice	274 (55.1%)
Pediatric practice	30 (6.0%)
Dermatological practice	28 (5.6%)
Ophthalmological practice	26 (5.2%)
Neurological practice	22 (4.4%)
Psychiatric/Psychosomatic practice	18 (3.6%)
Diabetology practice	12 (2.4%)
Cardiological practice	10 (2.0%)
Urological practice	8 (1.6%)
Endocrinological practice	7 (1.4%)
Surgical practice	7 (1.4%)
ENT practice	7 (1.4%)
Pneumological practice	6 (1.2%)
Orthopedic practice	4 (0.8%)
Gynecological practice	4 (0.8%)
Hematological/Oncological practice	4 (0.8%)
Gastroenterological practice	3 (0.6%)
Other	27 (5.4%)
No answer	1
**Patient information**	
Patient has a pharmacy customer file	
Yes	282 (59.4%)
No	186 (39.2%)
Unknown	7 (1.5%)
No answer	23
**Means of contact**	
Time of contact	11:27 am ± 2:31 (range 7:50 am–7:00 pm)
Contact through	
Pharmacist	299 (60.5%)
Pharmacy engineer	32 (6.5%)
Pharmaceutical–technical assistant	146 (29.6%)
Pharmaceutical–commercial assistant	1 (0.2%)
Other	16 (3.2%)
No answer	4
Contacted person in practice	
Physician	130 (36.2%)
Non-physician personnel	216 (60.2%)
Both physician and non-physician personnel	13 (3.6%)
No answer	139
Communication via *	
Telephone	416 (83.9%)
Email	27 (5.4%)
Telefax	60 (12.1%)
Postal mail	11 (2.2%)
In person	61 (12.3%)
Telematics-infrastructure (KIM)	5 (1.0%)
Other	3 (0.6%)
No answer	2
Number of contact attempts	1.9 ± 1.4 (range 1–10)

Note. Values represent n, percentage of valid cases (%), mean, standard deviation, and range. * = multiple responses possible, ENT = ear, nose, and throat, Non-physician personnel = employees working in the practice who are not physicians, e.g., medical assistants, nursing staff, medical technicians, but also students or administrative staff. KIM = Kommunikation im Medizinwesen/communication in healthcare. Pharmacy engineer = Apprenticeship program in the former German Democratic Republic (GDR), involving responsibilities falling between those of a pharmacy technician and a pharmacist; profession is being phased out.

**Table 3:** Reasons for establishing contact with practices.

n	498
Drug–drug interaction in general	16 (3.2%)
Drug–drug interaction between prescribed medications	9 (1.8%)
Drug–drug interaction between prescribed and self-medication	1 (0.2%)
Dosage/application in general	43 (8.6%)
Incorrect/unclear dosing	21 (4.2%)
Application route	7 (1.4%)
Health condition complicates intake	1 (0.2%)
Contraindication in general	9 (1.8%)
Due to co-morbidity	2 (0.4%)
Due to age	2 (0.4%)
Due to certain conditions (e.g., gender, pregnancy)	2 (0.4%)
Plausibility	22 (4.4%)
Double prescription	5 (1.0%)
Drug formulation	11 (2.2%)
Availability of medication	260 (52.2%)
Other (free text entry)	179 (35.9%)
Formal errors on prescription	54 (10.8%)
Clarifications or obtaining additional information	44 (8.8%)
Content errors on prescription	40 (8.0%)
Discussion of substitutions	19 (3.8%)
Drugs off the market or not prescribable	13 (2.6%)
Problems with electronic prescriptions	11 (2.2%)
Suspected prescription forgery	2 (0.4%)

Note. Multiple responses possible. Values represent n and percentage of valid cases (%).

**Table 4:** Outcome of and satisfaction with contact.

n	498
**Process and time required**	
Accessibility	
Contact made & reply received	415 (84.9%)
No one could be reached	52 (10.6%)
Practice called back later	6 (1.2%)
Contact only via electronic phone assistant	4 (0.8%)
Contact via patient	3 (0.6%)
Transfer of information via messenger	3 (0.6%)
Reply through e-mail	2 (0.4%)
Reply within a couple of days	2 (0.4%)
Assistant was sent to practice	2 (0.4%)
No answer	9
Duration of the conversation	
<5 min	350 (80.8%)
5–10 min	71 (16.4%)
11–15 min	5 (1.2%)
>15 min	7 (1.6%)
No answer	65
Duration of the entire case	
<60 min	231 (69.8%)
1–12 h	44 (13.3%)
13–24 h	13 (3.9%)
>24 h	43 (13.0%)
No answer	167
**Outcome of contact**	
Satisfaction with outcome of the contact (1 = not satisfactory, 10 = very satisfactory)	8.3 ± 2.8 (range 1–10)
Pleasantness of interaction (1 = very unpleasant, 10 = very pleasant)	8.7 ± 2.3 (range 1–10)
Acceptance of the pharmacy’s recommendation	
No	26 (6.5%)
Partially	30 (7.5%)
Yes	344 (86.0%)
No answer	98

Note. Values represent n, percentage of valid cases (%), mean, standard deviation, and range. Duration of the entire case = period from when a prescription is submitted until the issue is (not) resolved or patient care is (not) ensured.

**Table 5:** Multiple regression analysis predicting satisfaction with the outcome of the contact.

Predictor	B	SE B	β	R^2^
				0.390
Constant	6.225	0.817		
GP practice/Specialist practice	−0.329	0.244	−0.067	
Number of contact attempts	−0.235	0.100	−0.116 *	
Duration of the conversation	−0.443	0.270	−0.082	
Acceptance of the pharmacy’s recommendation	2.634	0.220	0.585 **	
Reason: Drug–drug interaction	−0.319	0.818	−0.021	
Reason: Dosage/application in general	−0.438	0.495	−0.052	
Reason: Contraindication in general	0.136	0.872	0.008	
Reason: Plausibility	−0.067	0.708	−0.005	
Reason: Availability of medication	−0.825	0.516	−0.168	
Reason: Other	−1.343	0.509	−0.262 *	

Note. Durbin-Watson = 1.560, * *p* < 0.005, ** *p* < 0.001.

**Table 6:** Multiple regression analysis predicting the duration of the entire case.

Predictor	Category	Estimate	SE	Wald	CI
Number of contact attempts		0.495	0.098	25.448 **	[0.3; 0.7]
Specialization of practice	GP	−0.672	0.302	4.953 *	[−1.3; −0.1]
	Specialist (Ref)	0			
Accessibility	Contact made & reply received	1.102	0.567	3.781	[−0.0; 2.2]
	No one could be reached (Ref)	0			
Reason: Drug–drug interaction	No	−0.430	1.008	0.182	[−2.4; 1.5]
	Yes (Ref)	0			
Reason: Dosage/application in general	No	0.298	0.678	0.194	[−1.0; 1.6]
	Yes (Ref)	0			
Reason: Contraindication in general	No	0.833	1.421	0.344	[−2.0; 3.6]
	Yes (Ref)	0			
Reason: Plausibility	No	−0.431	1.013	0.181	[−2.4; 1.6]
	Yes (Ref)	0			
Reason: Availability of medication	No	−0.408	0.643	0.401	[−1.7; 0.9]
	Yes (Ref)	0			
Reason: Other	No	−0.243	0.635	0.147	[−1.5; 1.0]
	Yes (Ref)	0			

Note. * *p* < 0.005, ** *p* < 0.001.

**Table 7:** Issues in the free text fields.

n	292
**Additional effort**	
Effort for pharmacy	280 (95.9%)
Not apparent from free text entry	12 (4.1%)
Effort for the patient	211 (72.3%)
Not apparent from free text entry	81 (27.7%)
Effort for practice	236 (80.8%)
Not apparent from free text entry	56 (19.2%)
**Impact on patient care**	
Patient care ensured	
Yes	191 (65.4%)
No	44 (15.1%)
Not apparent from free text entry	57 (19.5%)
Prescription	
As per original	34 (11.6%)
Alternative (preparation/dose/mode of action)	118 (40.4%)
No alternative available	17 (5.8%)
Not apparent from free text entry	123 (42.1%)
Prescription adjustment necessary	
Explicitly mentioned	97 (33.2%)
Not necessary/not specified	195 (66.8%)
**Interpersonal statements**	
Contact interaction	
Assessed positively	75 (25.7%)
Assessed negatively	94 (32.2%)
Assessed neutrally	123 (42.1%)
Not apparent from free text entry	0
Availability of the practice	
No availability	21 (7.2%)
Poor/long waiting time	31 (10.6%)
Good	2 (0.7%)
Not apparent from free text entry	238 (81.5%)
Need for clarification caused by	
Pharmacy	2 (0.7%)
Patient	13 (4.5%)
Practice	106 (36.3%)
Availability of medication	91 (31.2%)
Not apparent from free text entry	0

Note. Values represent n and percentage of valid cases (%).

## Data Availability

The data presented in this study are available on request from the corresponding author and are not publicly available due to privacy and ethical restrictions.

## Supplementary Materials

The following supporting information can be downloaded at: https://www.mdpi.com/article/10.3390/healthcare14121602/s1, Supplementary Material S1: Documentation form; Supplementary Material S2: Study evaluation; Supplementary Material S3: Drugs and medical products that were the reason for establishing contact (n = 468); Supplementary Material S4: Pharmacy’s evaluation of the study.

## Abbreviations

The following abbreviations are used in this manuscript:

ARMIN	Arzneimittelinitiative Sachsen-Thüringen
GP	General Practitioner/General Practice
ENT	Ear, nose, throat
KIM	Kommunikation im Medizinwesen (communication in healthcare)
ABDA	Bundesvereinigung Deutscher Apothekerverbände
