# Supplementary material for: Reasons and Outcomes of Pharmacy-Initiated Communication with Medical Practices—A Flashmob Study in Germany
Source: Healthcare (Basel). 2026 Jun 6;14(12):1602. doi: 10.3390/healthcare14121602 (PMC13299973; doi:10.3390/healthcare14121602)
Supplement: Supplementary file 1 [file healthcare-14-01602-s001.zip › healthcare-4282118-supplementary.pdf]

Supplementary material. Abbreviations used in supplementary material.

Note: Not listed alphabetically, but in the order in which they appear in Supplementary Material S1–S4:

GP practice	General practitioners' practice
ENT	Ear, nose, throat practice
PTA	Pharmaceutical technical assistant / Pharmacy technician
PCA	Pharmaceutical commercial assistant
MTA	Medical technical assistant / Medical technician
NSAIDs	Non-Steroidal Anti-Inflammatory Drugs
WHO	World Health Organization
COPD	Chronic Obstructive Pulmonary Disease
PPIs	Proton Pump Inhibitor

Supplementary material S1. Documentation form.

UNIVERSITÄT LEIPZIG
Medizinische Fakultät

– Study to record communication between pharmacies and medical practices –

Pharmacy details: ID

Documentation form for consultation with medical practices

1. Time of contact with the practice:
Date of contact: ________________________ Time of contact: ____________________________

2. Postal code of the practice contacted *(please refer to the prescription)*:
☐☐☐☐☐

3. Specialization of the practice contacted:
☐ GP practice [1] ☐ Neurology ☐ Urology ☐ Pneumology ☐ Gastroenterology
☐ Pediatrics ☐ Psychiatry/Psychosomatics ☐ Endocrinology ☐ Orthopaedics ☐ Other: _ _ _ _ _ _ _
☐ Dermatology ☐ Diabetology ☐ Surgical practice ☐ Gynecology
☐ Ophtalmology ☐ Cardiology ☐ ENT ☐ Hematology/Oncology

4. Contact was established by:
☐ Pharmacist ☐ Pharmacy engineer ☐ PTA ☐ PCA ☐ Other: _ _ _ _ _ _ _ _ _ _ _ _

5. Way of contact:
Please tick all that were used: ☐ Telephone ☐ Email ☐ Telefax ☐ Postal mail ☐ In person ☐ Other: _ _ _ _ _ _ _ _
Total number of contact attempts: ______

6. Reason for establishing contact *(multiple selection possible): There was a question or need for clarification regarding …*
☐ Drug-drug interaction(s)
- *between prescribed medications*
- *between prescribed and self-medication*

☐ Dosing/application
- *incorrect/unclear dosing*
- *application route*
- *health condition complicates intake*

☐ Contraindication(s)
- *due to co-morbitdity*
- *due to age*
- *due to certain conditions (e.g. gender, pregnancy)*

☐ Plausibility
- *double prescription*
- *drug formulation*

☐ Availability of medication

☐ Other: _ _ _ _ _ _ _ _ _ _ _ _ _ _ _ _ _

7. Names of prescribed medication/pharmaceuticals/medical product that were the reason for contact:
1. _ _ _ _ _ _ _ _ _ _ _ _ _ 2. _ _ _ _ _ _ _ _ _ _ _ _ _ 3. _ _ _ _ _ _ _ _ _ _ _ _ _ 4. _ _ _ _ _ _ _ _ _ _ _ _ _ _ _

8. If ‚Drug-drug interaction(s)' is selected under (7.): *The drug-drug interaction(s) became conspicuous through …*
☐ Note in the IT system ☐ Professional experience ☐ Other: _ _ _ _ _ _ _ _ _ _ _ _ _ _ _ _ _

9. The patient concerned has a pharmacy customer file:
☐ Yes ☐ No ☐ Unknown

10. How was the contact with the practice?
Accessibility: ☐ Contact made but no feedback ☐ No one could be reached[2] ☐ Contact made & reply received
Contact made with: ☐ Physician ☐ Non-physician personnel[3]

11. Satisfaction with outcome of the contact:

	1	2	3	4	5	6	7	8	9	10	
Not satisfactory	☐	☐	☐	☐	☐	☐	☐	☐	☐	☐	Very satisfactory

12. Was the pharmacy's recommendation accepted?
☐ Yes ☐ No ☐ Partially

13. Pleasantness of interaction:

	1	2	3	4	5	6	7	8	9	10	
Very unpleasant	☐	☐	☐	☐	☐	☐	☐	☐	☐	☐	Very pleasant

14. Processing time of the request:
Duration of the conversation (telephone/in person): ☐ < 5 min ☐ 5-10 min ☐ 11-15 min ☐ > 15 min
Duration of the entire case: ☐ < 60 min ☐ 1-12 hrs ☐ 13-24 hrs ☐ > 24 hrs

15. Comments/explanations on the contact and its outcome:
____
____
____
____

[1] GP practice concerns both general practitioners and internists working as general practitioners, [2] Documentation form does not need to be filled out further if selected,
[3] Non-physician personnel = physician assistant, MTA/medical technician, nurse, student

Supplementary material S2. Study evaluation.

Version: V01 (29.11.2023)

UNIVERSITÄT LEIPZIG

University of Leipzig, Faculty of Medicine,
Institute for General Practice,
Philipp-Rosenthal-Str. 55, 04103 Leipzig
ID «ID_Pharmacy»

Evaluation of the study
"Recording communication between pharmacies and medical practices"

Thank you very much for taking part in our study. We would like to use this evaluation form to find out more about how the study was integrated into your day-to-day business and how you rate the effort involved. Please complete all the questions and return the evaluation form to us in the enclosed envelope. Thank you very much!

Use of the documentation form in everyday business

**How often did the following problems occur during the study period?**	**Very often**	**Often**	**Some-times**	**Rarely**	**Never**
Not all pharmacy employees were sufficiently informed about the study.	☐	☐	☐	☐	☐
Staff shortages made it difficult to complete the documentation form.	☐	☐	☐	☐	☐
A large number of customers made it difficult to complete the documentation form.	☐	☐	☐	☐	☐
Finding the paper-based documentation form was not always possible in everyday business.	☐	☐	☐	☐	☐
Other problems: ________________					

**How do you rate the following aspects with regard to the application of the checklist?**	**Very good**	**Good**	**Suffi-cient**	**Poor**	**Very Poor**
User-friendliness of the documentation form in terms of comprehensibility.	☐	☐	☐	☐	☐
User-friendliness of the documentation form in terms of paper-based format.	☐	☐	☐	☐	☐
Time required to complete the documentation form.	☐	☐	☐	☐	☐
Personnel effort for completing the documentation form.	☐	☐	☐	☐	☐
Relevance of the documentation forms' content.	☐	☐	☐	☐	☐

**To what extent do you agree with the following statements in relation to the documentation form?**	**Totally agree**	**Rather agree**	**Partly**	**Rather disagree**	**Totally disagree**
The documentation form was easy to integrate into everyday business.	☐	☐	☐	☐	☐
I can imagine taking part in similar studies in the future.	☐	☐	☐	☐	☐

Other comments on the implementation of the study in your pharmacy:

____

Thank you very much for taking part in our study and completing the evaluation. We wish you and your team happy holidays and a healthy new year 2024!

Your Institute for General Practice of the University of Leipzig!

Supplementary material S3. Drugs and medical products that were the reason for establishing contact (n = 468).

**Category**	**Subcategory**	**n**	**%**
All cases with entries*		468	
Medications for the treatment of cardiological/ angiological/nephrological diseases and metabolic disorders	Antidiabetic drugs	68	14.5 %
	Antihypertensive drugs/medication for heart failure	55	11.8 %
	Lipid-lowering drugs	14	3.0 %
	Diuretics	6	1.3 %
	Other	7	1.5 %
Medications from the neurological/psychiatric spectrum	Antidepressants	26	5.6 %
	Neuroleptics	19	4.1 %
	Anticonvulsants	12	2.6 %
	Benzodiazepines	3	0.6 %
	Other	11	2.4 %
Anti-infective, antiseptic, and antiparasitic drugs	Antibiotics	55	11.8 %
	Antivirals	3	0.6 %
	Antiparasitics	2	0.4 %
	Other	4	0.9 %
Ophthalmic drugs		48	10.3 %
Analgesic drugs	NSAIDs and analgesics WHO I	22	4.7 %
	Opioids from WHO II	21	4.5 %
	Other	2	0.4 %
Medications from the pulmonology/ENT spectrum	Anti-asthmatics/anti-COPD drugs	15	3.2 %
	Antitussives	11	2.4 %
	Other	7	1.5 %
Endocrinologically and immunologically active drugs	Thyroid hormones	11	2.4 %
	Glucocorticoids	9	1.9 %
	Drugs for bone metabolism	8	1.7 %
Antithrombotic drugs	Anticoagulants	13	2.8 %
	Antiplatelet agents	3	0.6 %
Gastrointestinal drugs	Laxatives	5	1.1 %
	PPIs	5	1.1 %
	Antiemetics	2	0.4 %
	Other	4	0.9 %
Vaccines		10	2.1 %
Phytotherapeutics		6	1.3 %
Antineoplastic drugs		5	1.1 %
Homeopathic drugs		2	0.4 %
Other		54	9.4 %

Note. * = multiple responses possible.

Supplementary material S4. Pharmacy's evaluation of the study.

n	42					
Occurring problems during study	Very often	Often	Some-times	Rarely	Never	No answer
Uninformed pharmacy employees	1 (2.4%)	5 (11.9%)	9 (21.4%)	12 (28.6%)	15 (35.7%)	0
Staff shortages	8 (19.0%)	7 (16.7%)	14 (33.3%)	7 (16.7%)	6 (14.3%)	0
Large number of customers	4 (9.5%)	12 (28.6%)	15 (35.7%)	7 (16.7%)	4 (9.5%)	0
Availability of the paper-based documentation form	0 (0%)	1 (2.4%)	2 (4.8%)	8 (19.0%)	31 (73.8%)	0
Evaluation of the documentation form	Very good	Good	Sufficient	Poor	Very poor	No answer
Comprehensibility	26 (61.9%)	16 (38.1%)	0 (0%)	0 (0%)	0 (0%)	0
Paper-based format	24 (57.1%)	16 (38.1%)	2 (4.8%)	0 (0%)	0 (0%)	0
Time required	16 (38.1%)	20 (47.6%)	4 (9.5%)	1 (2.4%)	1 (2.4%)	0
Personnel effort	14 (33.3%)	22 (52.4%)	5 (11.9%)	0 (0%)	1 (2.4%)	0
Relevance of content	14 (34.1%)	23 (56.1%)	4 (9.8%)	0 (0%)	0 (0%)	1
How much do you agree with:	Totally agree	Rather agree	Partly	Rather disagree	Totally disagree	No answer
The documentation form was easy to integrate into everyday business	11 (26.2%)	26 (61.9%)	4 (9.5%)	1 (2.4%)	0 (0%)	0
I can imagine taking part in similar studies in the future	15 (36.6%)	21 (51.2%)	3 (7.3%)	2 (4.9%)	0 (0%)	1

Note. Values represent n and percentage of valid cases (%).
